# New Appliances and Things Medical

**Published:** 1903-08-15

**Authors:** 


### NEW APPLIANCES AND THINGS MEDICAL.

**[We shall be glad to receive at our Office, 28 & 29 Southampton Street, Strand, London, W.C., from the manufacturers, specimens of all new preparations and appliances which may be brought out from time to time.]**

### PEARSON'S ANTISEPTIC.

(PEARSON'S ANTISEPTIC CO., LTD., 254A HIGH HOLBORN LONDON, W.C.)

THIS preparation is a brown liquid forming a perfect emulsion with water. Experiments show that a 1 per cent. solution is capable of disinfecting the bacillus coli communis—a bacillus of high resisting power—in $2\frac{1}{2}$ minutes and the active staphylococcus pyogenes aureus in 5 minutes. The rapidity of action is often lost sight of in considering the potency of antiseptics. It is non-irritating in ordinary strengths and leaves a pleasant odour on the hands which is often desirable. It seems a useful addition to the already long list of these preparations.

### CHELTINE SOLUBLE MALTOSE FOOD.

(CHELTINE FOODS, LIMITED, CHELTINE WORKS, CHELTENHAM.)

THIS preparation when analysed is found to be entirely free from starch, cane sugar and cellulose fibre; this fact taken with its sterility and complete solubility shows it to be well adapted for the use of infants, while adults under many circumstances could take it with benefit. It is quickly prepared and palatable. In these days of hand fed infants it should prove of great value.

